# TNF differentially regulates ganglioside biosynthesis and expression in breast cancer cell lines

**DOI:** 10.1371/journal.pone.0196369

**Published:** 2018-04-26

**Authors:** Justine H. Dewald, Sumeyye Cavdarli, Agata Steenackers, Clément P. Delannoy, Marlène Mortuaire, Corentin Spriet, Maxence Noël, Sophie Groux-Degroote, Philippe Delannoy

**Affiliations:** 1 University of Lille, Structural and Functional Glycobiology Unit, UMR CNRS 8576, Lille, France; 2 University of Lille, Bio Imaging Center Lille, Lille, France; University of South Alabama Mitchell Cancer Institute, UNITED STATES

## Abstract

Gangliosides are glycosphingolipids concentrated in glycolipid-enriched membrane microdomains. Mainly restricted to the nervous system in healthy adult, complex gangliosides such as G_D3_ and G_D2_ have been shown to be involved in aggressiveness and metastasis of neuro-ectoderm derived tumors such as melanoma and neuroblastoma. GD3 synthase (GD3S), the key enzyme that controls the biosynthesis of complex gangliosides, was shown to be over-expressed in Estrogen Receptor (ER)-negative breast cancer tumors, and associated with a decreased overall survival of patients. We previously demonstrated that GD3S expression in ER-negative breast cancer cells induced a proliferative phenotype and an increased tumor growth. In addition, our results clearly indicate that Tumor Necrosis Factor (TNF) induced GD3S over-expression in breast cancer cells *via* NFκB pathway. In this study, we analyzed the effect of TNF on ganglioside biosynthesis and expression in breast cancer cells from different molecular subtypes. We showed that TNF up-regulated the expression of GD3S in MCF-7 and Hs578T cells, whereas no change was observed for MDA-MB-231. We also showed that TNF induced an increased expression of complex gangliosides at the cell surface of a small proportion of MCF-7 cells. These results demonstrate that TNF differentially regulates gangliosides expression in breast cancer cell lines and establish a possible link between inflammation at the tumor site environment, expression of complex gangliosides and tumor development.

## Introduction

**Fig 1:**
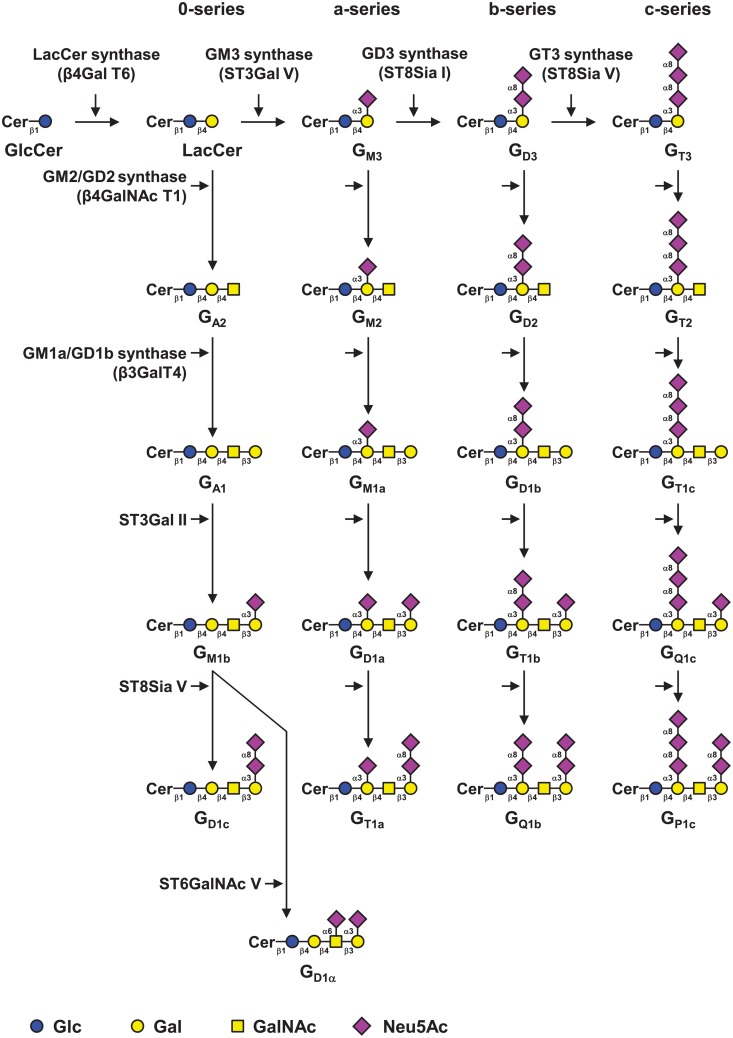
Biosynthesis of gangliosides. Gangliosides are classified in 4 series according to the number of sialic acid residues linked to lactosylceramide (LacCer) [22]. The 0-series gangliosides are directly synthesized from LacCer and the precursors of other series are synthesized by specific sialyltransferases: ST3Gal V (GM3 synthase), ST8Sia I (GD3 synthase) and ST8Sia V (GT3 synthase), respectively. The elongation of precursors is performed by the sequential action of N-acetyl-galactosaminyltransferase (β4GalNAc T1), galactosyltransferase (β3Gal T4) and sialyltransferases (ST3Gal II and ST8Sia V). Cer, ceramide. Adapted from [4].

Gangliosides define as subclass of acidic glycosphingolipids (GSL) carrying one or more sialic acid residues in the carbohydrate moiety. Gangliosides are essential compounds of the outer leaflet of the plasma membrane, where they interact with phospholipids, cholesterol, and transmembrane proteins to form glycolipid-enriched microdomains [1] in which they interact with signaling molecules including receptors tyrosine kinases and integrins, and regulate signal transduction pathways involved in cell adhesion, proliferation, and recognition processes, [2–4]. The carbohydrate moiety of gangliosides is synthesized in the Golgi apparatus by specific glycosyltransferases (GT) and gangliosides are classified in four series according to the number of sialic acid residues linked to the lactosylceramide (Fig 1). Changes in ganglioside composition are observed between human tissues, complex gangliosides with two or more sialic acid residues being normally restricted to the nervous system [5,6]. Changes in the structure of gangliosides can also occur under pathological conditions [7–9] and a neo-expression of disialogangliosides such as G_D2_ and G_D3_ is observed in several cancers from neuroectoderm origin including melanoma and neuroblastoma, in which they play a key role in invasion and metastasis [10], and disialogangliosides are attractive targets for cancer immunotherapy [11,12].

In breast cancer, complex gangliosides G_D3_ and 9-O-acetyl-G_D3_ have been reported to be over-expressed in about 50% of invasive ductal breast carcinoma [13] and the GD3 synthase (GD3S) gene displayed higher expression among estrogen receptor negative breast cancer tumors [14], associated with poor pathohistological grading and a decreased free survival of patients [15]. We previously demonstrated that the expression of GD3S in breast cancer cells induced a proliferative phenotype and increased tumor growth due to the constitutive activation of c-Met receptor by G_D2_ ganglioside [16–18]. We also demonstrated that GD3S gene expression is up-regulated by TNF *via* the NFκB pathway and that estradiol repressed GD3S expression in estrogen receptor (ER) positive breast cancer cells by preventing NFκB nuclear translocation [19]. Moreover, G_D2_ ganglioside was recently identified as a new breast cancer stem cells specific marker [20].

Given the critical role of both G_D2_ ganglioside and inflammation in breast cancer aggressiveness [21], and in order to provide a general overview of the effect of inflammatory cytokines on ganglioside biosynthesis, we examined the effect of TNF on the expression of the main ganglioside-specific GT genes as well as cell surface gangliosides in breast cancer cells from different molecular subtypes.

## Materials and methods

### Antibodies

Anti-G_M3_ mAb GMR6 (mouse IgM), anti-G_M2_ mAb MK1-16 (mouse IgM) and anti-G_D1b_ GGR12 (mouse IgG3) were purchased from AMS Biotechnology (Abingdon, UK). Anti-G_D3_ mAb R24 (mouse IgG3) and anti-G_D2_ mAb 14.G2a (mouse IgG2a) were purchased from Abcam (Cambridge, MA, USA). Fluorescein isothiocyanate (FITC)-conjugated cholera toxin B subunit from *Vibrio cholerae* used for G_M1a_ expression analysis was from Sigma-Aldrich (Saint-Quentin Fallavier, France). Alexa Fluor^®^ 488 donkey anti-mouse IgG (H+L) and Alexa Fluor^®^ 488 anti-mouse IgM (μ-chain) were purchased from Molecular Probes Invitrogen (Cergy Pontoise, France).

### Cell culture

The human breast cancer cell lines MCF-7 and MDA-MB-231 were obtained from LGC standards (Molsheim, France) and the American Type Cell Culture Collection (Rockville, MD, USA), respectively. The Hs578T human breast cancer cell line [23] was kindly provided by Dr Van Slambrouck (Department of Chemistry and Biochemistry, South Dakota State University, Brookings, SD 57007, USA). Cells were routinely grown in monolayer cultures in Dulbecco’s Modified Eagle Medium (DMEM) with 4.5 g/L glucose, 2 mM L-glutamine, supplemented with 10% fetal calf serum (FCS) and 100 μg/mL penicillin-streptomycin (Lonza, Verviers, Belgium) at 37°C in an atmosphere of 5% CO_2_. Cells were treated with 40 ng/mL TNF (Eurobio AbCys, Paris, France) during 12–24 h before qPCR analysis or during 48 h for other experiments.

### RNA Extraction, cDNA Synthesis and Quantitative real-time Polymerase Chain Reaction (qPCR)

Total RNA was extracted from the different cell lines using the Nucleospin RNA II kit (Macherey-Nagel, Düren, Germany). The amount of extracted RNA was quantified using a DeNovix DS-11 spectrophotometer (DeNovix Inc., Wilmington, DE, USA) and the purity of the RNA was checked by the ratio of absorbance at 260 nm *vs*. 280 nm. Total RNA was subjected to reverse transcription using the Maxima First Strand cDNA Synthesis Kit (ThermoFisher Scientific, France) according to the protocol provided by the manufacturer. PCR reactions were performed using 2X SYBR^®^ Green Universal QPCR Master Mix (ThermoFisher Scientific). Primer sequences (Eurogentec, Seraing, Belgium) used for the PCR reactions are given in Table 1. *HPRT* gene was used to normalize the expression of transcripts of interest. PCR reactions were performed using the Mx3005p Quantitative System (Stratagene) as previously described [16]. PCR conditions were as follows: 95°C for 30 s, Tm °C for 60 s, 72°C for 20 s (40 cycles). The analysis of amplification was performed using the Mx3005p software and relative quantification was performed using the method described by Pfaffl that takes in account the efficiency of each sequence amplification [24]. Student’s *t*-test was used for statistical analysis. A *p*-value < 0.05 was considered statistically significant.

**Table 1:** Primer pairs used for qPCR experiments.

Gene	Glycosyltransferase	Sense primer Antisense primer	Tm (°C)
***HPRT***		5’-GCCAGACTTTGTTGGATTTG-3’ 5’-CTCTCATCTTAGGCTTTGTATTTTG-3’	58
***ST3GAL5***	GM3 synthase	5’-ATCGGTGTCATTGCCGTTGT-3’ 5’-TTCATAGCAGCCATGCATTGA-3’	51
***ST8SIA1***	GD3 synthase	5’-GCGATGCAATCTCCCTCCT-3’ 5’-TTGCCGAATTATGCTGGGAT-3’	60
***B4GALNT1***	GM2/GD2 synthase	5’-CAGCGCTCTAGTCACGATTGC-3’ 5’-CCACGGTAACCGTTGGGTAG-3’	51
***B3GALT4***	GM1/GD1b synthase	5’-TATGTGCTGTCAGCGTCTGCT-3’ 5’-ACAAAGACATCCTCTAATGGGAGAA-3’	60

### Extraction and preparation of glycosphingolipids

Cultured cells were washed twice with ice-cold PBS and detached from T175 flasks with cell dissociation non-enzymatic solution. Cells were sonicated on ice in 200 μL of water. The resulting material was dried under vacuum and sequentially extracted by CHCl_3_/CH_3_OH (2:1, v/v), CHCl_3_/CH_3_OH (1:1, v/v) and CHCl_3_/CH_3_OH/H_2_O (1:2:0.8, v/v/v). Supernatants were pooled, dried and subjected to a mild saponification in 0.1 M NaOH in CHCl_3_/CH_3_OH (1:1) at 37°C for 2 h and then evaporated to dryness [25]. Samples were reconstituted in CH_3_OH/H_2_O (1:1, v/v) and applied to a reverse phase C_18_ cartridge (Waters, Milford, MA, USA) equilibrated in the same solvent. After washing with CH_3_OH/H_2_O (1:1, v/v), GSL were eluted by CH_3_OH, CHCl_3_/CH_3_OH (1:1, v/v) and CHCl_3_/CH_3_OH (2:1, v/v). The elution fraction was dried under nitrogen stream prior to structural analysis.

### Mass spectrometry analysis of GSL

Prior to mass spectrometry analysis, GSL were permethylated according to Ciucanu and Kerek [26]. Briefly, compounds were incubated 2 h in a suspension of 200 mg/mL NaOH in dry DMSO (300 μL) and CH_3_I (200 μL). The methylated derivatives were extracted in CHCl_3_ and washed several times with water. The reagents were evaporated and the sample was dissolved in CHCl_3_ in the appropriate dilution. MALDI-MS and MS/MS analyses of permethylated GSL were performed on 4800 Proteomics Analyzer (Applied Biosystems, Framingham, MA, USA) mass spectrometer, operated in the positive reflectron mode. For MS acquisition, 5 μL of diluted permethylated samples in CHCl_3_ were mixed with 5 μL of 2,5-dihydroxybenzoic acid matrix solution (10 mg/mL dissolved in CHCl_3_/CH_3_OH (1:1, v/v)). The mixtures (1 μL) were then spotted on the target plate and air dried. MS survey data comprises a total of 50 sub-spectra of 1500 laser shots. Peaks observed in the MS spectra were selected for further MS/MS. CID MS/MS data comprises a total of 100 sub-spectra of 3000 laser shots. Two or more spectra can be combined post-acquisition with mass tolerance set at 0.1 Da to improve S/N ratio. The potential difference between the source acceleration voltage and the collision cell was set to 1 kV and argon was used as collision gas.

### Flow cytometry analysis

Cells were plated in 6-well plates (2.5 × 10^5^ cells/well). The next day, cells were treated with 40 ng/mL TNF for 48 h. Cells were detached by trypsin and incubated for 30 min at 4°C with anti-gangliosides mAbs: anti-G_M3_ (1:100), anti-G_M2_ (1:100), anti-G_D3_ (1:100), anti-G_D2_ (1:100) or anti-G_D1b_ (1:100), all diluted in PBS containing 0.5% bovine serum albumin (BSA; Sigma-Aldrich, Lyon, France). After washing with PBS/0.5% BSA, cells were incubated for 30 min on ice with Alexa Fluor^®^ 488 anti-mouse IgM or IgG (dilution 1:500 in PBS/0.5% BSA). Controls were performed using secondary antibodies alone. To analyze G_M1a_ expression, cells were incubated with a FITC-conjugated cholera toxin B subunit from *Vibrio cholerae* (1:1000) for 30 min at 4°C. Controls were performed using non-stained cells. Cells were then subjected to flow cytometry analysis using a FACScalibur flow cytometer from Becton Dickinson (Le-Pont-de-Claix, France).

### Immunofluorescence staining

MCF-7 cells were plated on glass coverslips (ThermoFisher Scientific) in 6-well plates (2 × 10^5^ cells/well). The next day, cells were treated with 40 ng/mL TNF for 48 h. Cells were then washed with DPBS and fixed 15 min in 4% paraformaldehyde at room temperature. After washing, cells were permeabilized in 0.5% Triton X-100 and blocked 1 h in a blocking buffer containing 2% BSA, 2% FCS and 0.2% gelatin in PBS. Cells were incubated for 1 h at room temperature with anti-G_M3_ (1:100), anti-G_M2_ (1:100), anti-G_D3_ (1:100), anti-G_D2_ (1:100) or anti-G_D1b_ (1:50) antibodies diluted in blocking buffer. After three washes with PBS, cells were incubated 1 h with appropriated secondary antibodies (dilution 1:1000 in blocking buffer) and with the FITC-conjugated cholera toxin B subunit from *Vibrio cholerae* for G_M1a_ analysis (1:4000). Cells were washed with PBS then with deionized water, and finally mounted in Fluorescent mounting medium (Dako, Carpenteria, CA, USA). The nuclei were stained with DAPI. Slides were examined under a Zeiss LSM 700 confocal microscope. The image acquisition characteristics were the same throughout the different conditions to ensure the comparability of the results and to allow the comparison of the fluorescence levels. For statistical analysis, images of ≥ 100 cells for each condition were collected based on the DAPI signal and were then analyzed for G_D3_ and G_D2_ expression using an automated algorithm on ImageJ software. Anova was used for statistical analysis. A *p*-value < 0.05 was considered statistically significant.

## Results

### MS analysis of GSL of breast cancer cells

**Fig 2:**
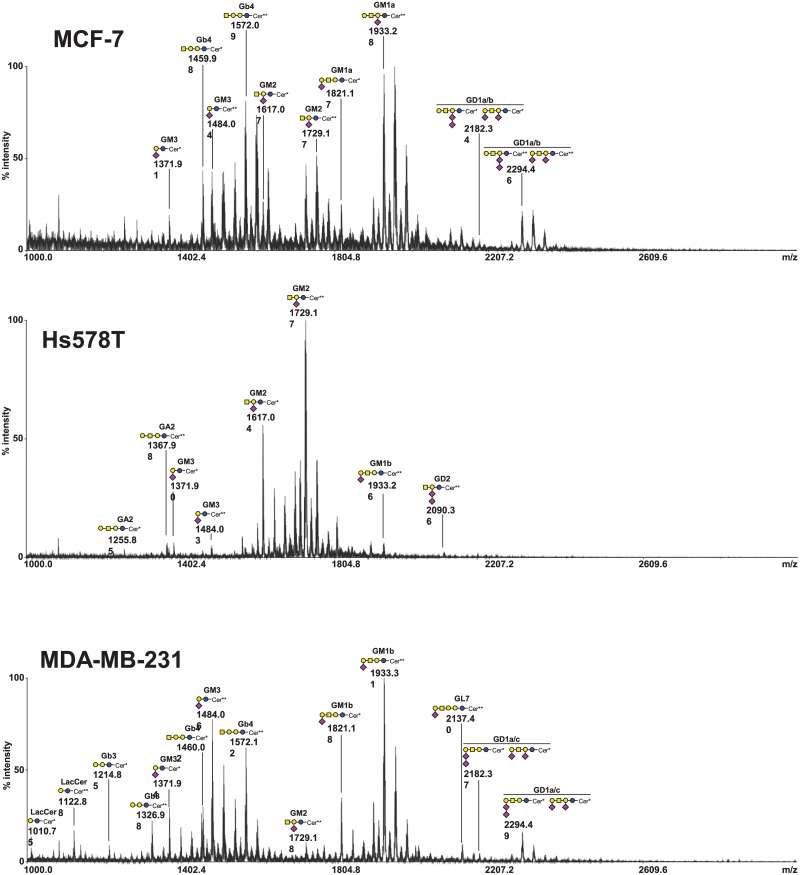
Mass spectrometry (MALDI-TOF) analysis of permethylated GSL isolated from MCF-7, Hs578T and MDA-MB-231 cells. Blue circle, Glc; yellow circle, Gal; yellow square, GalNAc; purple diamond, Neu5Ac. Only masses corresponding to N-palmitoyl- (C16:0) or N-lignoceroyl- (C24:0) 2-amino-4-octadecene-1,3-diol (sphingosine) are indicated. Cer*: Ceramide d18:1/C16:0; Cer**: Ceramide d18:1/C24:0.

The composition of total GSL was determined by MALDI-TOF mass spectrometry after permethylation in three commonly used breast cancer cell lines, i.e. the luminal-A MCF-7 cells, the basal-like MDA-MB-231 cells, whose ganglioside composition was already analyzed [16], and the basal-like Hs578T cells, whose ganglioside profile appeared to be quite different from the two other cell lines (Fig 2 and Table 2). Two ceramide isoforms are commonly expressed in human tissues due to the substitution of the sphingosine moiety by palmitic acid C16:0 or lignoceric acid C24:0. In MCF-7 cells, an additional ceramide isoform was observed with a cerotic acid C26:0 linked to sphingosine. As expected, MCF-7 essentially expressed a-series gangliosides, mainly G_M1a_, whereas MDA-MB-231 cells mainly expressed G_M1b_ as confirmed by fragmentation of permethylated GSL using MALDI-TOF/TOF mass spectrometry (data not shown). G_M3_ and G_M2_ were also expressed in both cell lines but in lower amounts. Both cell lines also expressed low amounts of disialogangliosides. Signals at *m/z* 2182.34 and 2294.46 corresponding to G_D1_ isomers with 3 hexoses, one N-acetylhexosamine and 2 N-acetylneuraminic acid residues, were confirmed as and mixture of G_D1a_ (90%) and G_D1b_ (10%) in MCF-7 cells and a mixture of G_D1a_ (40%) and G_D1c_ (60%) in MDA-MB-231 cells. Both cell lines also expressed neutral GSL from globo-series such as G_b3_ and G_b4_. These results are in agreement with previous published data [18,27]. The GSL composition of Hs578T cells was never previously determined and appeared relatively simpler compared to the two other cell lines. Hs578T cells mainly expressed the monosialoganglioside G_M2_ but very low amounts of G_M3_ and G_M1b_. Neutral GSL from globo-series such as G_b3_ and G_b4_ was also almost absent and only a low amount of G_A2_ was detected. Finally, Hs578T cells also expressed a low amount of the disialoganglioside G_D2_ as indicated by the signal at *m/z* 2090.1.

**Table 2:** Compositional assignments of singly charged sodiated molecular ions [M + Na]^+^, observed in MALDI-TOF mass spectrometry spectra of permethylated GSL from MCF-7, Hs578T and MDA-MB-231 cells.

Fatty acids	GSL	Calculated mono-isotopic molecular masses	MCF-7	Hs578T	MDA-MB-231
16:0	LacCer	1010.7485	-	-	1010.75
24:0	LacCer	1122.8743	-	-	1122.88
16:0	G_b3_	1214.8483	-	-	1214.85
24:0	G_b3_	1326.9741	-	-	1326.98
16:0	G_A2_	1255.8748	-	1255.85	-
24:0	G_A2_	1367.9736	-	1367.98	-
16:0	G_M3_	1371.9222	1371.91	1371.90	1371.94
24:0	G_M3_	1484.0480	1484.04	1484.03	1484.06
16:0	G_b4_	1459.9746	1459.98	1459.96	1460.02
24:0	G_b4_	1572.1004	1572.09	1572.08	1572.12
16:0	G_M2_	1617.0485	1617.07	1617.04	1617.10
24:0	G_M2_	1729.1743	1729.17	1729.17	1729.18
16:0	G_D3_	1733.0958	-	-	-
24:0	G_D3_	1845.2216	-	-	-
16:0	G_M1a/b_	1821.1483	1821.17 (G_M1a_)		1821.18 (G_M1b_)
24:0	G_M1a/b_	1933.2741	1933.28 (G_M1a_)	1933.26 (G_M1b_)	1933.31 (G_M1b_)
16:0	G_D2_	1978.2222	-	1978.24	-
24:0	G_D2_	2090.3480	-	2090.36	-
16:0	G_T3_	2094.2695	-	-	-
24:0	G_T3_	2206.3953	-	-	-
16:0	G_L7_	2025.2481	-	-	-
24:0	G_L7_	2137.3739	-	-	2137.40
16:0	G_D1a/b_	2182.3219	2182.34 (G_D1a/b_)	-	2182.37 (G_D1a/c_)
24:0	G_D1a/b_	2294.4477	2294.46 (G_D1a/b_)	-	2294.49 (G_D1a/c_)

Only masses corresponding to N-palmitoyl- (C16:0) or N-lignoceroyl- (C24:0) 2-amino-4-octadecene-1,3-diol (sphingosine) are indicated. Assignments were confirmed by mass spectrometry fragmentation of permethylated GSL by MALDI-TOF/TOF (data not shown). LacCer, lactosylceramide; Gb, globoside.

### Effect of TNF on the expression of ganglioside GTs in breast cancer cell lines

**Fig 3:**
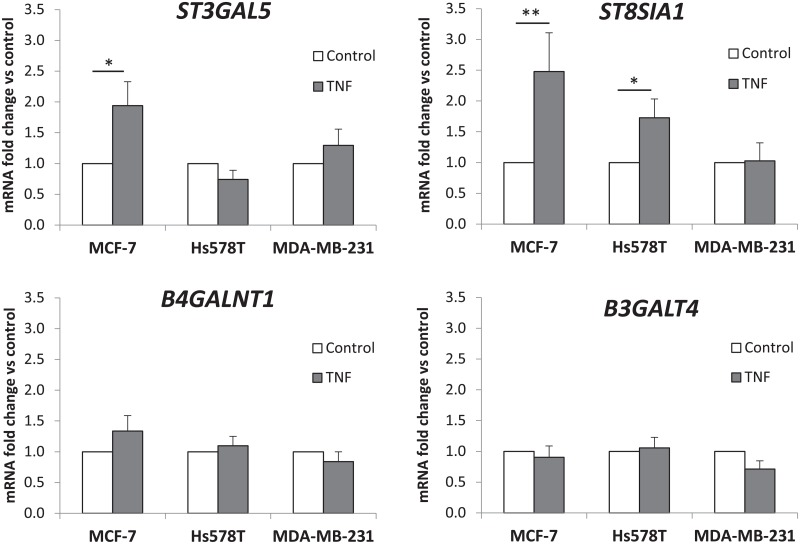
Effect of TNF on GTs expression in MCF-7, Hs578T and MDA-MB-231 cell lines. Cells were treated with 40 ng/mL TNF for 12-24h. *ST3GAL5*, *ST8SIA1*, *B4GALNT1* and *B3GALT4* mRNA expression was determined by qPCR. Results were normalized to the expression of HPRT and reported to the expression of GTs in non-treated cells (control). Each bar represents the mean +/- S.E.M. of n ≥ 3 experiments. *: *p* < 0.05, **: *p* < 0.01 *vs*. untreated (control).

The effect of TNF on the expression of glycosyltransferase genes involved in ganglioside biosynthesis was determined by qPCR for the three cell lines. The mRNA levels for each GT gene, i.e. *ST3GAL5* encoding the GM3 synthase, *ST8SIA1* encoding the GD3 synthase, *B4GALNT1* encoding the GM2/GD2 synthase and *B3GALT4* encoding the GM1/GD1b synthase, were normalized to the expression of *HPRT* and reported to the expression of GT genes in non-treated cells (Fig 3). Our results confirmed the increased expression of *ST8SIA1* after TNF treatment in MCF-7 and Hs578T as previously reported [19] but no change in the expression of *ST8SIA1* was observed in MDA-MB-231 cells. We also confirmed by qPCR the expression of *TNFR1*, the gene encoding the TNF receptor 1, in the three cell lines (data not shown). TNF treatment also increased the expression of *ST3GAL5* in MCF-7 but no significant change of *ST3GAL5* expression was observed in both Hs578T and MDA-MB-231 cells. In parallel, no significant change of *B4GALNT1* and *B3GALT4* gene expression under TNF treatment was observed in the three cell lines. These results indicate that TNF treatment could increase the biosynthesis of b-series gangliosides, especially in MCF-7 cells, by increasing the expression of two essential GT genes (*ST3GAL5* and *ST8SIA1*) whereas TNF had no effect on the expression of these genes in MDA-MB-231 cells.

### Flow cytometry analysis of gangliosides at the cell surface of breast cancer cells

**Fig 4:**
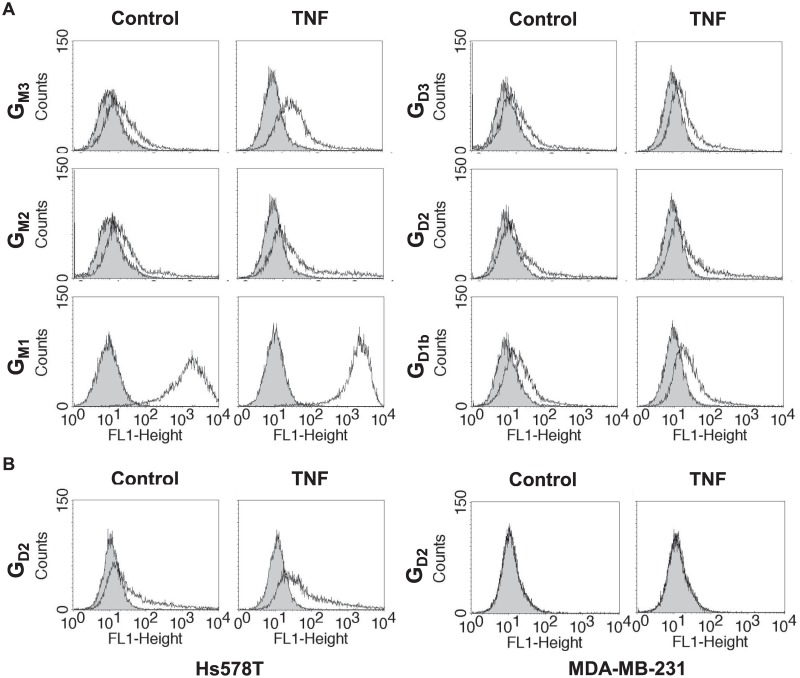
Flow cytometry analysis of the effect of TNF on gangliosides expression at the surface of breast cancer cell lines. MCF-7 (**A**), Hs578T and MDA-MB-231 (**B**) cells were treated with 40 ng/mL TNF for 48h. Control and treated cells were incubated with anti-G_M3_, G_M2_, G_D3_, G_D2_ or G_D1b_ specific antibodies and revealed with and Alexa Fluor^®^ 488 anti-mouse IgG or IgM. G_M1a_ ganglioside was revealed with the FITC-conjugated cholera toxin B subunit from *Vibrio cholerae*. The grey peaks correspond to negative controls with anti-mouse IgG or IgM secondary antibody alone or with non-stained cells as control for G_M1_ signal. Results are from one experiment representative of 3 independent experiments.

To establish the ganglioside profile of control and TNF-treated cells, we analyzed the composition of gangliosides at the cell surface using specific anti-ganglioside mAbs or cholera toxin B subunit for G_M1a_ detection. The results presented in Fig 4A showed that MCF-7 cells expressed a limited number of gangliosides of the a-series, mainly G_M1a_. Low amounts of G_M3_ and G_M2_ were also detected confirming the results of MS analysis. Moreover, we were able to confirm the presence of low amount of G_D1b_ but also of G_D3_ and G_D2_ in a very limited subpopulation of MCF-7 cells, whereas these b-series gangliosides were not detected by MS analysis. In agreement with the increased expression of GM3 synthase transcripts observed by qPCR, G_M3_ and G_M2_ expression was increased at the cell surface of MCF-7 cells after TNF treatment. Importantly and also in agreement with qPCR analysis, the subpopulation expressing G_D3_ and G_D2_ also appeared to be higher in MCF-7 treated cells than in control cells. In parallel, we analyzed the expression of gangliosides at the surface of Hs578T cells. Interestingly we could only detect an increased expression of G_D2_ in TNF-treated cells (Fig 4B). Finally, MDA-MB-231 cells presented a ganglioside pattern similar to MCF-7 cells (data not shown), and no change of ganglioside expression was observed at their cell surface after TNF treatment, in agreement with qPCR analysis. These results indicate that the increased expression of GT genes induced by TNF in both MCF-7 and Hs578T cells led to an increased expression of disialoganglioside G_D2_ at the cell surface.

### Immunocytochemistry analysis of gangliosides in MCF-7 cells

**Fig 5:**
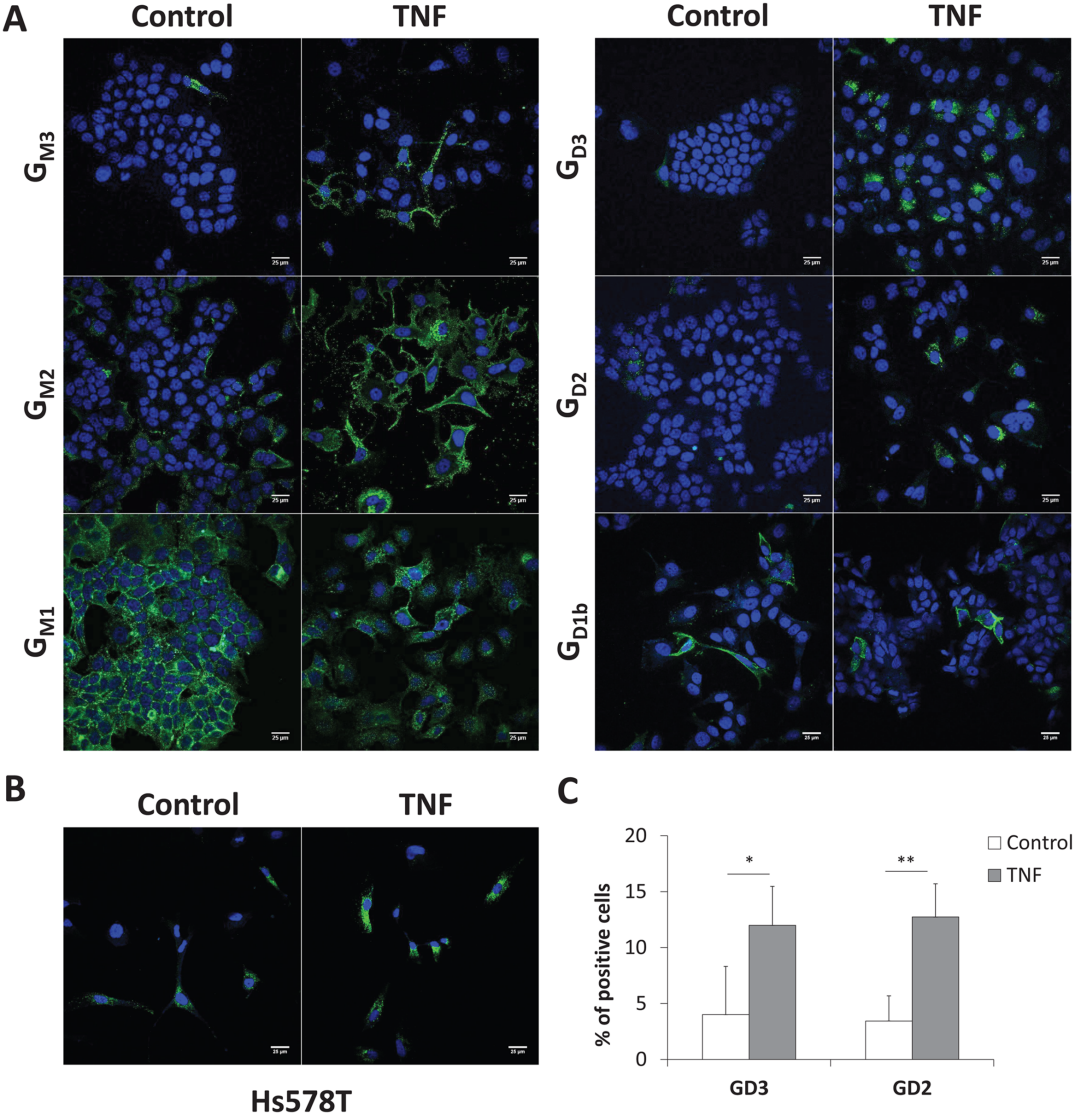
Immunocytochemistry and confocal microscopy analysis of gangliosides expression. MCF-7 cells (**A**) and Hs578T cells (**B**) were treated with 40 ng/mL TNF for 48h. Control and treated cells were incubated with anti-G_M3_, G_M2_, G_D3_, G_D2_ or G_D1b_ specific antibodies and revealed with Alexa Fluor^®^ 488 conjugated anti-mouse IgG or IgM. G_M1a_ ganglioside was revealed with the FITC-conjugated cholera toxin B subunit from *Vibrio cholerae*. The nuclei were counter stained with DAPI. Bars: 25 μm. For statistical analysis (**C**), images of ≥ 100 MCF-7 cells for each condition were collected based on the DAPI signal and were analyzed for G_D3_ and G_D2_ expression using an automated algorithm on ImageJ software. *: *p* < 0.05, **: *p* < 0.01 *vs*. untreated (control).

In order to confirm the results obtained by flow cytometry, control and TNF-treated MCF-7 were analyzed by immunofluorescence and confocal microscopy. As shown in Fig 5A, G_M1a_ was highly expressed at the plasma membrane of MCF-7 cells, while lower amounts of G_M3_ and G_M2_ could be observed. The expression of G_D3_ and G_D2_ was also very low in control MCF-7 cells. After 48 h of incubation with TNF, G_M3_ and G_M2_ levels at the plasma membrane were higher, whereas no significant change was observed for G_M1a_, which remained highly expressed after TNF treatment. Finally, the analysis of G_D2_ expression in Hs578T cells allowed us to confirm the increased expression of G_D2_ at the cell surface of treated cells observed by flow cytometry (Fig 5B). To quantify variations in ganglioside expression in MCF-7 cells after TNF treatment, we analyzed the population expressing G_D3_ and G_D2_ using an automated algorithm on ImageJ software. For each condition, images of ≥ 100 control and treated-cells were collected based on the DAPI signal for total cell counting. In the control cell population, G_D3_ and G_D2_ expressing cells were estimated at 4.0 ± 4.0% and 3.4 ± 2.3% of total cells, respectively, whereas TNF treatment extended these populations to 12 ± 3.5% and 12.7 ± 3.0% (Fig 5C).

## Discussion

Complex gangliosides from b-series are oncofetal markers of human tumors of neuroectoderm origin such as melanoma, glioblastoma and neuroblastoma where they play a functional role in tumor growth and metastasis by mediating cell proliferation, migration and angiogenesis [7]. Previous studies have shown that the expression of ganglioside-specific GTs was also altered in breast cancer tumors. In particular, the GD3S was shown to display higher expression among ER-negative breast cancer tumors [14], associated with a lower survival rate of patients [15]. In parallel, the gangliosides G_D3_, as well as its derivatives 9-O-acetyl-G_D3_ and 9-O-acetyl-G_T3_ are over-expressed in about 50% of invasive ductal carcinoma, whereas they show a very restricted expression in normal breast tissues, [13]. In that context, we previously showed that the ectopic expression of the GD3S in breast cancer cells induced the accumulation of b- and c-series gangliosides at the cell surface together with the acquisition of a proliferative phenotype and enhanced tumor growth [16,17], due to the specific and constitutive activation of c-Met receptor in the absence of ligand, and subsequent activation of the PI3K/Akt and Erk/MAPK pathways in G_D2_ expressing breast cancer cells [18]. Ganglioside G_D2_ was also identified as a putative marker of CD44^hi^CD24^lo^ breast cancer stem cells (CSC) capable of initiating tumors, and several GT genes involved in G_D2_ biosynthesis (*ST3GAL5*, *B4GALNT1*, and *ST8SIA1*) are highly expressed in CSC [20]. Moreover, the induction of epithelial–mesenchymal transition (EMT) in transformed human mammary epithelial cells dramatically increased GD3S as well as G_D2_ expression [28], confirming the role of GD3S and G_D2_ in breast cancer progression.

Chronic inflammation in the tumor micro-environment is known to play an important role in cancer progression and NFκB was proposed as an important actor of inflammation-driven malignancy [21]. Several GTs were previously described to be regulated by TNF [29]. As an example, we showed that TNF up-regulates *ST3GAL4* expression through an intronic ATF2-responsive element, resulting in sialyl-Lewis^x^ antigen over-expression in lung epithelial cells [30]. Concerning GT genes involved in ganglioside biosynthesis, a functional NFκB binding site at -777/-762 pb upstream the ATG was shown to be essential for *ST8SIA1* transcription in melanoma cells [31] and inflammatory cytokines including TNF and IL-6 enhanced GD3S gene expression in melanocytes [32]. We also reported that TNF enhanced GD3S expression in ER-negative breast cancer cells *via* NFκB pathway [19]. However, the effect of TNF on the expression of other ganglioside-specific GT genes in breast cancer cells was still unknown. In the present study, we examined the effect of TNF on the expression of the main ganglioside-specific GT genes as well as ganglioside expression in three breast cancer cells from different molecular subtypes. The most potent effect of TNF was observed for MCF-7 cells, in which TNF significantly enhanced the expression of genes encoding the GD3S (*ST8SIA1*) and also the GM3 synthase (*ST3GAL5*) that synthesizes G_M3_, the precursor of both G_M2_ and G_D3_. In contrast, the expression of the other ganglioside-specific GTs genes *B4GALNT1* and *B3GALT4* was not significantly modified after TNF treatment, showing that TNF controls the expression of enzymes involved in the first steps of a- and b-series biosynthesis in MCF-7 cells. It was previously reported that exposure of mouse mammary carcinoma cells to TGF-β and TNF induced EMT and generated cells with a CSC phenotype [33]. It was also reported that the mRNA levels for *ST3GAL5*, *ST8SIA1*, but also for *B4GALNT1* and *ST3GAL2* were increased in human breast CSC model induced through EMT [34]. The ER-positive MCF-7 cells belongs to luminal breast cancer subtype with an epithelial phenotype [35] and the changes in the expression of GT genes that we observe in this study could be therefore associated with TNF-induced EMT of MCF-7 cells.

TNF treatment also significantly enhanced the expression of GD3S gene in Hs578T but the expression of the other GTs was not modified. The triple negative Hs578T cells were previously shown to over-express GD3S when treated by TNF [19]. In contrast with observations made in MCF-7 cells, TNF had no effect on the expression of the GM3 synthase. Surprisingly, no change in the expression of all GTs genes tested was observed for the triple negative MDA-MB-231 cells, which are of the same molecular subtype than Hs578T cells, and known to express the TNF receptor 1 (TNFR1) [36]. We also confirmed the expression of TNFR1 by qPCR in the three cell lines (data not shown). These results indicate that inflammation differently modulates the expression of ganglioside-specific GT genes in breast cancer cells.

The effect of TNF treatment on cell surface ganglioside expression was in parallel analyzed by flow cytometry and confocal microscopy. As expected, an increased expression of G_M3_ and G_M2_ was observed in TNF-treated MCF-7 cells that fitted well with the increased of GM3 synthase gene expression. G_M2_ is the precursor for the biosynthesis of G_M1a_ and the high and maybe saturated expression of G_M1a_ in control conditions could also explain the accumulation of G_M2_ under TNF treatment. In renal carcinoma cells, TNF increased the expression of G_M2_ by enhancing the mRNA level of *B4GALNT1* encoding the GM2/GD2 synthase, and resulted in T cell death and immune dysfunction [37]. As a result of the increased expression of GD3S, a significant increase of G_D2_ was observed by flow cytometry in both TNF-treated MCF-7 and Hs578T cells and confocal microscopy analysis allowed us to confirm the expression of G_D2_ in about 13% of MCF-7 cells. Confocal microscopy also revealed an intracellular labeling of G_D3_ presumably in the Golgi apparatus, showing the presence of the G_D2_ precursor in TNF treated cells. These results demonstrate for the first time that TNF can modify the expression of gangliosides and increase G_D2_ expression at the cell surface of MCF-7 and Hs578T breast cancer cells. It was previously shown that TNF/TGFβ treatment of mouse mammary carcinoma cells induced EMT and generated breast cancer stem cells with a claudin-low molecular subtype characterized by the expression of mesenchymal and stem cell-associated markers, and associated with a poor prognosis [33]. According to the role of complex gangliosides, especially G_D2_, in breast cancer cells EMT and aggressiveness, the increased expression of G_D2_ in breast cancer cells under TNF treatment could establish a link between the presence of pro-inflammatory cytokines at the tumor site environment, expression of complex gangliosides and EMT, resulting in more aggressive cells with increased tumorigenicity and increased resistance to treatment.

## Abbreviations

BSA: bovine serum albumin
CSC: cancer stem cells
DAPI: 4',6-diamidino-2-phénylindole
DMEM: Dulbecco’s Modified Eagle Medium
DMSO: dimethyl sulfoxide
EMT: epithelial–mesenchymal transition
ER: estrogen receptor
FCS: fetal calf serum
FITC: fluorescein isothiocyanate
GD3S: GD3 synthase
GSL: glycosphingolipids
GT: glycosyltransferase
HPRT: hypoxanthine phosphoribosyltransferase
MALDI-TOF: Matrix Assisted Laser Desorption Ionisation—Time of Flight
MS: mass spectrometry
PBS: phosphate buffered saline
qPCR: quantitative real-time polymerase chain reaction
TNF: tumor necrosis factor
TNFR1: tumor necrosis factor receptor 1
